# Massive sublingual hematoma secondary to anticoagulant therapy complicated by a traumatic denture: a case report

**DOI:** 10.1186/1752-1947-6-105

**Published:** 2012-04-10

**Authors:** Anchal Puri, Muzzammil A Nusrath, Deepak Harinathan, Jamie Lyall

**Affiliations:** 1 Department of Oral & Maxillofacial Surgery, James Cook University Hospital, Middlesbrough, UK

## Abstract

**Introduction:**

Sublingual hematoma secondary to excessive anticoagulation is a rare but potentially fatal condition, and few cases have been documented in the literature.

**Case presentation:**

We report the case of a 73-year-old Caucasian woman who attended our Accident and Emergency department with massive sublingual hematoma causing superior displacement of the tongue. The condition was found to be the result of an elevated international normalized ratio, further complicated by a traumatic mandibular denture.

**Conclusions:**

In summary, we recommend the immediate reversal of anticoagulation therapy on admission of patients with severe sublingual hematoma. We further advise surgical decompression/drainage if required and to continue meticulous monitoring. In all cases of early recognition of sublingual hematoma, prompt medical treatment and continuous clinical monitoring is essential, and may prevent the need for a surgical airway procedure.

## Introduction

Anticoagulant therapy is a commonly prescribed regimen most frequently utilizing warfarin. Oral anticoagulants work by depressing the activity of coagulation factors such as thrombin, prothrombin, and factors VII, IX, and X. There are several recognized indications for the use of oral anticoagulants. These include deep venous thrombosis, pulmonary embolism, vascular thromboembolism, mechanical valve replacement, cerebral venous thrombosis, certain hypercoaguable states, transient ischemic attack and completed cerebral ischemia (ischemic stroke) which is secondary to atrial fibrillation.

Hemorrhagic complications of warfarin therapy are well known. Such complications usually appear in the genitourinary, gastrointestinal, retroperitoneal and intracranial areas, with few reports involving the upper airway. In relation to these cases, airway compromise often stems from retropharyngeal and laryngeal hematomas, and only occasional reports state sublingual hematoma formation as a result of deficient coagulation [1,2].

We report the case of a massive sublingual hematoma with superior displacement of the tongue. The condition was found to be the result of an elevated international normalized ratio (INR), further complicated by a traumatic mandibular denture.

## Case presentation

**Figure 1:**
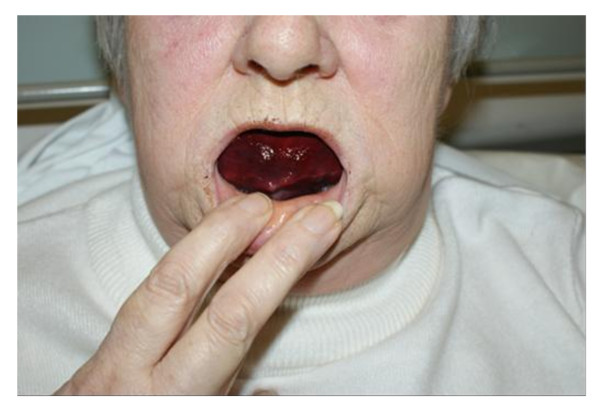
**Sublingual hematoma displacing the Wharton's ducts and floor of the mouth superiorly**.

A 73-year-old Caucasian woman was rushed to our Accident and Emergency department after discovering a large swelling in her mouth on awakening. She had intermittent bleeding from the site with mild pain. Her history consisted of a recently constructed set of complete acrylic dentures made one week previously by her general dental practitioner. It was reported these had been slightly uncomfortable ever since she had received them. Later questioning further revealed that our patient had worn her dentures continuously, even at night, but had not yet returned to her dentist for a follow-up. Her medical history was significant for rheumatic fever as a child, resulting in valvular heart disease for which our patient had mitral and aortic mechanical valve replacements in 1991 and 2002, respectively. Warfarin 5 mg had been initiated to obtain a target INR between 2.5 and 3.5. Our patient also had hypothyroidism, which was being treated with thyroxine replacement therapy. On arrival to the hospital, her full blood count was found to be within the normal range, however her INR was recorded as 5.5. A clinical examination revealed a large, soft, dark red swelling involving the anterior region of the floor of her mouth, indicative of sublingual hematoma. The Wharton's duct of the submandibular gland was distinctly visible on opening (Figure 1). As a result of the hematoma, the tongue was displaced superiorly and there was mild limitation of tongue movement. Our patient was acyanotic at the time of admission and on general examination there was no signs of stridor or major airway restriction. Further flexible endoscopic examination did not reveal any edema or obstruction within the pharynx or larynx.

On palpitation there were firm, bilateral swellings noted in the submandibular and submental regions. Her mouth opening was approximately 7 mm on first presentation.

Some hoarseness of her voice was appreciated, but our patient's vital signs were stable. Oxygen (5 L/minute) was delivered through a nasal cannula and our patient was advised to sit in an upright position at this time.

Our patient was transferred to our Acute Assessment Unit where close monitoring of her airway was undertaken. Warfarin was stopped and 2000 IU of Beriplex^® ^prothrombin complex concentrate supplemented with 2 mg intravenous vitamin K was administered to reverse the anticoagulation. In addition, 100 mg of intravenous hydrocortisone was provided to assist in decreasing any associated edema. At approximately two hours post-transfusion the INR was corrected to 1.0. Although the airway was patent on arrival, the hematoma was seen to be increasing in size during this time and our patient was at risk of airway occlusion.

Immediate decompression of the sublingual space was performed under general anesthetic. A conscious intubation was carried out using a fiber-optic flexible nasal endoscope. Had this not have been successful a surgical cricothyroidotomy or tracheostomy would have been required to facilitate the surgery. After successful intubation, an initial horseshoe-shaped incision was made followed by blunt dissection above the mylohyoid muscle. The lingual nerve and Wharton's duct of the submandibular gland were identified and preserved. Bipolar diathermy was carried out, as well as packing of the area with absorbable oxidized cellulose agent (Surgicel^®^) to prevent any further bleeding. As the nasopharynx and hypopharynx were found to be clear with no obvious obstructions, a clinical decision was made to extubate our patient after surgery.

Our patient was transferred to our Intensive Care Unit where she remained for four days. She was given 1 mg/kg (70 mg) of subcutaneous low-molecular-weight heparin (LMWH), which continued daily for 10 days. On day three, our patient's warfarin treatment was restarted at a dose of 2 mg initially in combination with LMWH. Daily checks of her INR were carried out and as a result, her warfarin dosage was gradually increased accordingly. During this time our patient experienced a few occasional bouts of mild bleeding from the surgical site that were easily stopped with 5% tranexamic acid mouthwash. Ecchymosis later appeared over the anterior surface of the neck.

Adequate healing was soon noted in the area of the surgical site, with no evidence of further bleeding. Our patient was discharged on the 12th hospital day with a therapeutic range INR of 2.8, achieved with 4 mg of warfarin. She was advised to discontinue wearing her dentures until any necessary adjustments had been made by her general dental practitioner. At review two months later she is doing well with no signs of recurrence.

## Discussion

Treatment of an acute sublingual hematoma requires early recognition and intervention. Initially it is important to assess, and if necessary, secure the airway. If anticoagulation is the cause this needs to be reversed [1].

It is not uncommon for warfarin therapy to instigate complications within the body. However, hemorrhages caused by a warfarin overdose usually appear in the genitourinary, gastrointestinal, retroperitoneal and intracranial areas and few reports have implied warfarin therapy as a cause of hematoma of the upper airway [2]. In our patient's case, it was obvious that the hematoma was principally a result of our patient's abnormal INR, which was above the ideal therapeutic level. However, due to her recently constructed dentures it is also likely these potentially exacerbated the condition.

Dentures are among the most widely used and accepted prostheses available; some renowned complications associated with them include allergy to the denture material, chronic stomatitis and other occasional complications such as blockage of the submandibular duct orifices [3]. A minority of cases in the literature have indicated a causative factor for sublingual hematoma arising from trauma alone without any anticoagulation significance, such as after oral surgical procedures [4]. It however appears to be rare for a traumatic denture to be the single cause of a hematoma of this description, with only one previous case being reported [3].

Therefore, our diagnosis in this case concerned the formation of a sublingual hematoma resulting from excessive oral anticoagulation complicated by a secondary trauma from denture use.

In 1976, Lepore described what is known as a 'pseudo-Ludwig's phenomenon', a condition caused by deranged anticoagulation resulting in spontaneous bleeding in the sublingual and submaxillary spaces [5]. This can cause elevation of the tongue and floor of the mouth, thus instigating respiratory distress and eventually complete upper airway obstruction. Due to the potentially life-threatening nature of this condition, rapid reversal of anticoagulation is required.

In previous cases, reversal of anticoagulant therapy has often been carried out with fresh frozen plasma (FFP) in combination with vitamin K [1,2]. Beriplex^® ^prothrombin complex concentrate was used in our patient's case. Beriplex is produced by fractionation of pooled plasma and contains factors II, VII, IX and X. It has been found more favorable in achieving rapid and complete reversal of an abnormal INR in an emergency. FFP has the disadvantage of being required in large volumes and is available at a slow rate, as it needs to thaw. It also needs to be group specific. FFP is however, less expensive than prothrombin complex concentrate and is argued to have a lower risk of thromboembolism [6,7]. Intravenous vitamin K and fresh frozen plasma are therefore generally used for less severe bleeding, and provide a more gradual reversal.

It is also worth acknowledging the necessity of surgical drainage versus non-surgery in the case of the sublingual hematoma. The majority of previously described cases have found spontaneous resolution of the hematoma once coagulation is normalized [4]. In our patient's case however, due to the progressive nature of the hematoma, surgical decompression was deemed mandatory.

Earlier reports have also advised the use of a surgical airway via cricothyroidotomy or tracheostomy [1,2]. Fortunately for our patient, repeated flexible nasal endoscopies were reassuring, allowing for more conservative management without the additional morbidity of a surgical airway procedure. Whether or not such an intervention is required should always be based on the clinical presentation. If there is any indication of severe obstruction or edema within the pharynx or larynx during the endoscopic examination no hesitation should be made to establish a surgical airway immediately.

## Conclusions

In summary, we recommend the immediate reversal of anticoagulation therapy, ideally with Beriplex^® ^prothrombin complex concentrate supplemented with vitamin K, on the admission of patients with severe sublingual hematoma [2]. We further advise surgical decompression/drainage if required and to continue meticulous monitoring.

Sublingual hematomas can quickly develop into a life-threatening condition, and early recognition, prompt medical treatment and continuous clinical monitoring may prevent the need for a surgical airway procedure.

## Consent

Written informed consent was obtained from the patient for publication of this case report and any accompanying images. A copy of the written consent is available for review by the Editor-in-Chief of this journal.

## Competing interests

The authors declare that they have no competing interests.

## Authors' contributions

AP and MAN were the major contributors to writing the manuscript. DH was involved in the care of our patient and revised the case report. JL was responsible for the medical and surgical care of our patient. All authors read and approved the final manuscript.
